# Structural Studies of Inhibitors with Clinically Relevant Influenza Endonuclease Variants

**DOI:** 10.1021/acs.biochem.3c00536

**Published:** 2024-01-08

**Authors:** Alysia J. Kohlbrand, Ryjul W. Stokes, Banumathi Sankaran, Seth M. Cohen

**Affiliations:** † Department of Chemistry and Biochemistry, University of California, La Jolla, California 92093, United States; ‡ The Berkeley Center for Structural Biology, Advanced Light Source, Lawrence Berkeley National Laboratory, Berkeley, California 94720, United States

## Abstract

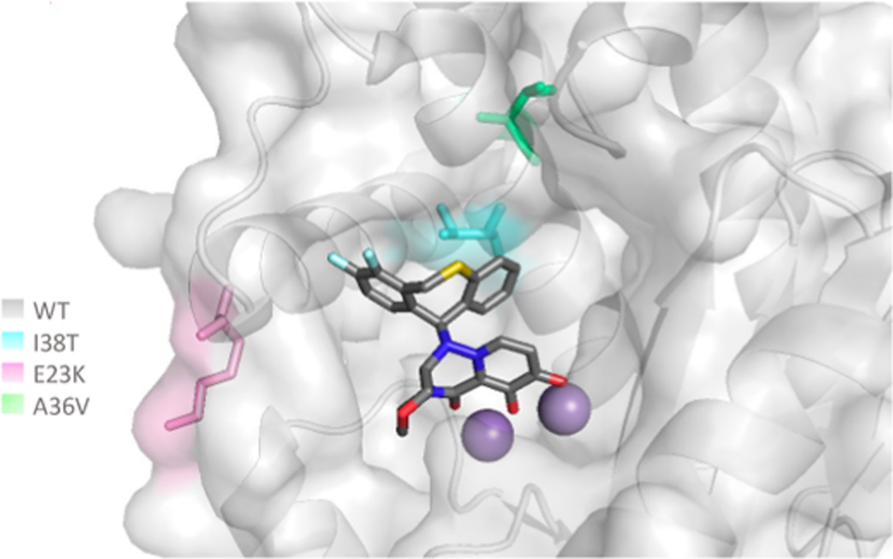

Vital to the treatment of influenza is the use of antivirals such as Oseltamivir (Tamiflu) and Zanamivir (Relenza); however, antiviral resistance is becoming an increasing problem for these therapeutics. The RNA-dependent RNA polymerase acidic N-terminal (PA_N_) endonuclease, a critical component of influenza viral replication machinery, is an antiviral target that was recently validated with the approval of Baloxavir Marboxil (BXM). Despite its clinical success, BXM has demonstrated susceptibility to resistance mutations, specifically the I38T, E23K, and A36 V mutants of PA_N_. To better understand the effects of these mutations on BXM resistance and improve the design of more robust therapeutics, this study examines key differences in protein–inhibitor interactions with two inhibitors and the I38T, E23K, and A36 V mutants. Differences in inhibitor binding were evaluated by measuring changes in binding to PA_N_ using two biophysical methods. The binding mode of two distinct inhibitors was determined crystallographically with both wild-type and mutant forms of PA_N_. Collectively, these studies give some insight into the mechanism of antiviral resistance of these mutants.

## Introduction

The influenza virus is responsible for a significant burden of illness, causing approximately 35.5 million cases, 500,000 hospitalizations, and 35,000 deaths in the United States during the 2018/19 season alone.^1^ Children and elderly populations are particularly vulnerable to complicated cases of influenza and make up the largest percentage of hospitalizations and deaths.^2^ During the COVID-19 pandemic, nonpharmaceutical interventions (NPIs) such as stay-at-home orders, masking, social distancing, and increased disinfection measures were put into practice in public spaces to prevent the spread of SARS-CoV-2. This also led to a substantial decrease of global influenza infections through the 2020/21 and 2021/22 seasons,^3^ which has consequences for the annual reformulation of the influenza vaccine. Reformulation is heavily dependent on data from prior infectious seasons of circulating strains to predict the most effective vaccine composition for the coming influenza season.^3^ Therefore, there were less data to predict the optimal 2022/23 vaccines, which explains the substantial resurgence of influenza observed for the 2022/23 season.^3,4^ The use of antivirals for influenza is a crucial second line of defense, especially during times of resurgence, epidemics, and pandemics.

**Figure 1:**
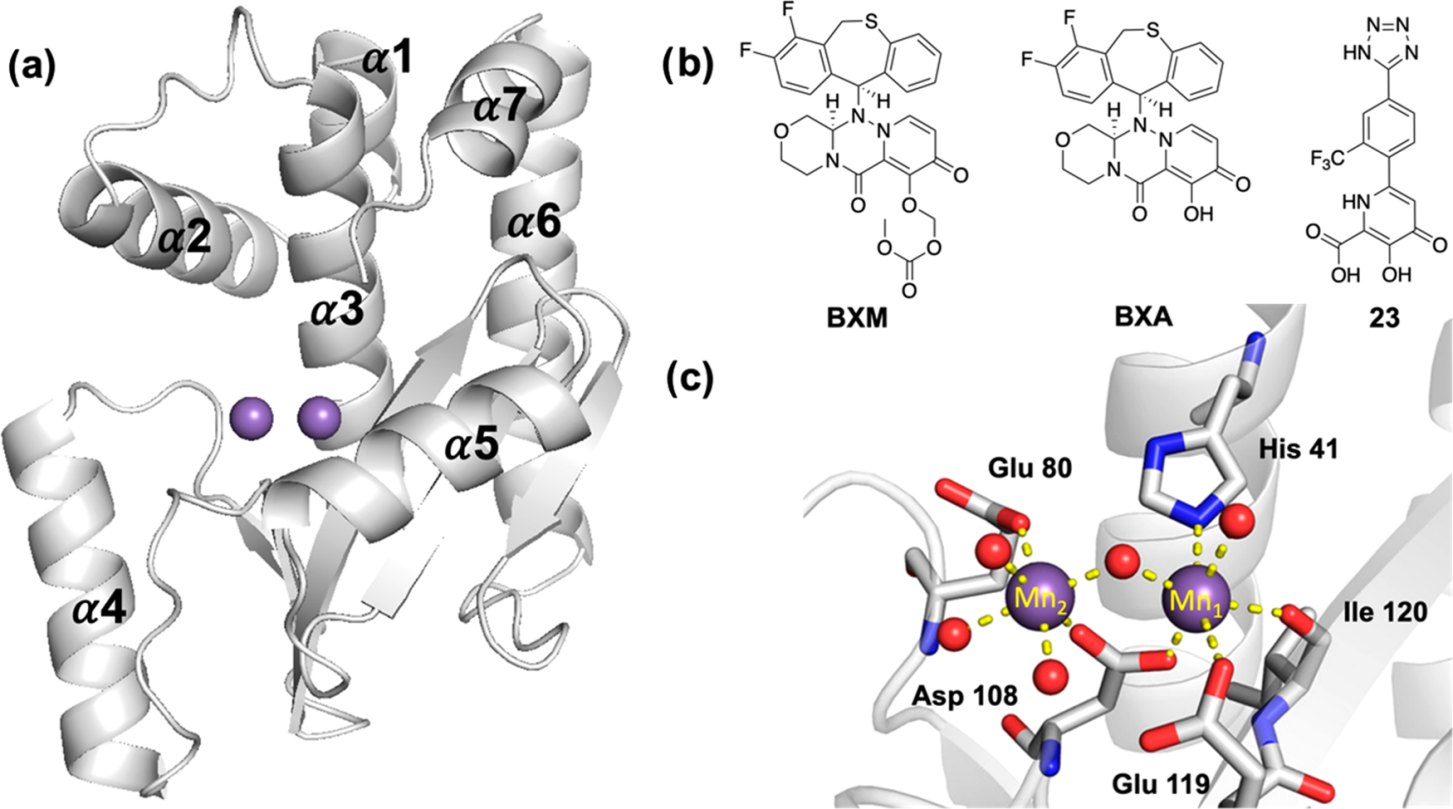
(a) Wild type (WT) PA_N_ endonuclease with all α helices labeled (PDB: 8T5Z). (b) Baloxavir marboxil (BXM), its active metabolite baloxavir acid (BXA), and compound **23**. (c) PA_N_ active site with Mn^2+^ ions shown and coordinating residues labeled. The protein backbone (gray) is shown as a gray ribbon. Mn^2+^ ions and water molecules are shown as purple and red spheres, respectively. Residues coordinating the Mn^2+^ ions are shown in sticks.

Vital to the treatment of influenza is the use of neuraminidase inhibitors (NAIs) such as Oseltamivir (Tamiflu) and Zanamivir (Relenza). Although antiviral resistance remains low for these medications, resistance has been on a steady rise and there is an urgent need for new medications with novel mechanisms of action.^5^ Baloxavir marboxil (BXM) and its active metabolite baloxavir acid (BXA) is a first-in-class inhibitor targeting the influenza virus polymerase PA N-terminal endonuclease domain (PA_N_) that was approved in Japan and the United States in 2018 (Figure 1).^6^ The RNA-dependent RNA polymerase of the influenza virus cannot synthesize its own 5′-mRNA cap, which is necessary for eukaryotic translation. To overcome this, the polymerase engages in a unique “cap snatching” mechanism, in which the polymerase subunit PB2 captures host pre-mRNA and PA_N_ cleaves 10–13 nucleotides to serve as a primer for viral mRNA synthesis by the PB1 subunit.^7,8^ PA_N_ is a metalloenzyme that is essential for viral replication, is highly conserved, and is not naturally occurring in humans, making it an attractive antiviral target.^6^ BXM has been shown to reduce fever in an average of 24 h and is an oral monotherapy that can be taken at the time of diagnosis, which ensures higher patient compliance than medications taken over several days. Despite its clinical success, BXM has also demonstrated a high susceptibility to treatment-emergent resistance and viral rebound.^6,9^

In a resistance monitoring study following the 2014 approval of BXM in Japan, many treatment-emergent resistance mutations were found. The highest change in susceptibility and most prevalent resistance mutations found were in isoleucine 38 with I38T/M/F/L/N/S mutations. I38T has been reported to decrease the efficacy of the BXM by 30- to 50-fold.^6,10^ Other emergent mutations of interest are E23K and A36 V mutations, like I38T, which were identified as resistance mutations resulting from BXM treatment.^6^ The probability of widespread transmission of these variants depends on the replication capacity and onward transmission. Recent transmissibility studies in animal models of these mutations suggest that the I38T mutation on its own has a reduced fitness compared to WT viruses; however, I38T in combination with other stabilizing mutations can recover.^11^ These fast emergent resistance mutations highlight the need for additional members of this class of antivirals and a better understanding of the mechanisms of resistance for rational drug design.

Unlike NAI resistance mechanisms that have been studied extensively for over 20 years, the resistance landscape of PA_N_ endonuclease inhibitors is largely unknown, and the monitoring of resistance markers (I38X and E23X) is still in its infancy. Several structural studies of the I38T variant with BXA have been published, and it is largely agreed that the reduced activity of BXA is due to less favorable van der Waals packing and induced fit changes within the PA_N_ active site.^6,12−14^ To date, the structures of E23K and A36V have not been published; however, in silico studies for the E23K/G mutations hinted at the possibility of the destabilization of the α2 helix (Figure 1) and disruption of other important interactions for RNA recognition by BXA.^15−17^ Several other potent inhibitors for PA_N_ endonuclease have been developed. A group of hydroxypyridinone heterocycle compounds have been reported to bind to the PA_N_ endonuclease active site metals with a similar metal-binding pharmacophore (MBP) to BXA.^18,19^ A majority of these compounds are known to make van der Waals contacts with Ile38 and could be at a similar disadvantage as BXA.^19^ Of these hydroxypyridinone heterocycles, compound **23** is among the most potent and is included here to examine the effects of BXA resistance mutants on binding to an alternative inhibitor type (Figure 1).

In the present study, seven co-crystal structures of BXA and a second PA_N_ inhibitor (**23**) with WT, I38T, E23K, and A36V PA_N_, as well as the apo (ligand-free) structures of WT, I38T, and E23K constructs are reported. The E23K and A36 V structures are the first reported structural data for these mutants and may aid in studies to develop a structure–activity relationship (SAR) for these variants. The structural data obtained are complimented by inhibitor affinity determined by two independent biophysical methods to quantify the impact of the changes in binding observed in the co-crystal structures.

## Results and Discussion

### Biophysical Evaluation

Due to the high affinity of the inhibitors used in this study, determining the inhibitory activity by measuring IC_50_ values using conventional FRET-based enzymatic assays is not particularly informative, as many inhibitors will simply display activity at concentrations below the sensitivity threshold of the assay. Therefore, inhibitor binding in this study was evaluated using biophysical methods to determine binding constants (*K*_d_) and changes in the thermal stability (*T*_M_) of the enzyme. Although *K*_d_ is fundamentally different, and not directly comparable to an IC_50_ value, overall trends between *K*_d_ and IC_50_ are typically comparable.^20^

Spectral shift methods (SpS) are based on the well-known phenomenon that organic fluorophores can report changes in their chemical environment via perturbations in their emission spectrum, e.g., changes in their overall fluorescence intensity or *l*_max_ (red or blue spectral shifts). In the studies performed here, fluorescence was recorded at two wavelengths (650 and 670 nm), and the change in emission intensity between the 670/650 nm fluorescence signal was monitored as a function of ligand concentration (see the SI for experimental details). This method allows for the determination of binding constants (*K*_d_) over a large dynamic range from low nanomolar (nM) to high millimolar (mM) values.^21,22^ In this study, purified PA_N_ endonuclease was used (see the SI for experimental details).^19^ Purified PA_N_ is known to bind substrates and inhibitors with poorer affinity than the complete PA subunit or heterotrimer polymerase complex, which may explain why the *K*_d_ values reported here (see below) are weaker than reported elsewhere.^23^ Using this method, binding of BXA to WT PA_N_ proved to be the tightest binding interaction measured in this study (343 nM, Table 1). Binding of BXA to the I38T mutant showed a 27-fold reduction in affinity that is consistent with previously reported studies of BXA affinity for this mutant (30- to 50-fold reduction).^6^ There are fewer data available on the binding of BXA to the E23K mutant, but the SpS experiments showed an even larger 79-fold reduction in binding affinity.

**Table 1:** Dissociation Constants (*K*_d_, μM) and Relative Fold Change in Binding Affinity (FC) of PA_N_ Endonuclease Variants with BXA and **23**

	BXA		**23**	
	*K*_d_ (μM)	relative affinity	*K*_d_ (μM)	relative affinity
WT	0.343 ± 0.106		277 ± 30.6	
I38T	9.3 ± 4.2	27-fold	384 ± 19.4	1.4-fold
E23K	26.1 ± 11.2	79-fold	328 ± 26.6	1.2-fold

Compound **23** is known to have a poorer affinity than BXA for WT PA_N_. This was verified by SpS, with **23** giving a *K*_d_ value of 277 μM for WT PA_N_ (∼660-fold weaker than BXA).^6,12,19^ Although the affinity of **23** is not as high as BXA for WT PA_N_, the relative change in affinity of **23** for the I38T and E23K variants is much smaller compared to BXA (<2-fold for **23**, compared to 27- and 79-fold for BXA). This suggests that inhibitors that access different parts of the active site may be less susceptible to mutations generated against BXA. However, it is important to note that BXA still has a substantially greater absolute affinity for I38T and E23K when compared to compound **23** (Table 1). Of note, the A36V construct was found to denature and precipitate out of solution when left at room temperature for prolonged periods of time, including the time required for incubation in the SpS assay. Thus, the A36V construct was not stable enough for the determination of an accurate *K*_d_ value by the SpS method and is not reported here.

The thermal stability of a protein generally increases upon formation of favorable protein–ligand contacts, with tighter-binding interactions typically resulting in larger changes in thermal shift (Δ*T*_M_) compared to those of an apo (ligand-free) control. Differential scanning fluorometry (DSF) experiments were performed with both BXA and compound **23** with WT PA_N_ and all three mutants. WT, I38T, and E23K PA_N_ displayed an average melting temperature of between 58 and 61 °C, while A36 V proved to be much less thermally stable with a melting temperature of 50–52 °C (Table 2). DSF experiments were performed at 200 μM for both BXA and **23**. A concentration of 200 μM is sufficient for the low *K*_d_ values of BXA, but it is lower than the *K*_d_ values for compound **23** (≥200 μM). To compare these two compounds with a sufficient excess of compound, an additional measurement at 1 mM was performed for compound **23**. As described below, the melting temperatures for experiments run with 1 mM compound **23** showed an expected shift to higher values. BXA bound to WT PA_N_ produced the largest Δ*T*_M_ at 22.9 °C (Table 2). The difference in Δ*T*_M_ for BXA bound to I38T compared to WT was ∼8 °C lower with a Δ*T*_M_ of 14.9 °C. This indicates the interaction between BXA and I38T is not as stable as WT. This finding is consistent with previous studies that reported Δ*T*_M_ melting value between the WT and I38T mutants with BXA to be between 8 and 10 °C.^6,12^ Binding of BXA to the E23K mutant produced a Δ*T*_M_ of 19.8 °C, which is <3 °C difference when compared to binding to WT PA_N_. This suggests some loss in stability when BXA is bound to E23K when compared to WT PA_N_, but still results in significant stabilization of the E23K mutant enzyme. Interestingly, a larger change in Δ*T*_M_ was observed when BXA is bound to the E23K mutant versus the I38T mutant, contradicting the SpS experiments, which show the opposite trend in affinity. This may indicate that the E23K mutation promotes dissociation of BXA without affecting protein stability. This E23K mutant may increase flexibility of α2 encouraging BXA to dissociate and preventing new binding events. The melting temperature of the A36V mutant is significantly lower than its counterparts, adding to the evidence that the construct itself is not as stable as the other mutations in this study.^24^ However, the Δ*T*_M_ of the A36 V mutant with BXA is 21.7 °C, nearly identical to that of BXA with WT, suggesting the binding of BXA to the A36 V mutation is strong despite the instability of this mutant.

**Table 2:** Thermal Melting Temperature Change of PA_N_ Endonuclease Variants with BXA and **23** (Δ*T*_M_, °C)^a^

	DMSO	BXA (200 μM)	**23** (200 μM)	**23** (1 mM)
	*T*_M_ (°C)	Δ*T*_M_ (°C)	Δ*T*_M_ (°C)	Δ*T*_M_ (°C)
WT	58.7 ± 0.11	22.9 ± 0.08	17.8 ± 0.10	19.6 ± 0.16
I38T	59.6 ± 0.08	14.9 ± 0.04	12.9 ± 0.09	15.6 ± 0.28
E23K	61.5 ± 0.08	19.8 ± 0.06	10.2 ± 0.08	14.3 ± 0.09
A36V	51.3 ± 0.20	21.7 ± 0.11	16.4 ± 0.14	17.1 ± 0.12

a Experiments with BXA and **23** were averaged from eight independent measurements.

Compound **23** bound to WT PA_N_ showed a Δ*T*_M_ of 17.8 °C at 200 μM and 19.6 °C at 1 mM (Table 2), which is <5 °C less than BXA bound to WT and consistent with the large difference in *K*_d_ values between these compounds (Table 1). The Δ*T*_M_ for **23** bound to I38T was found to be 12.9 °C at 200 μM and 15.6 °C at 1 mM, which is around 4 °C less than the WT. Like BXA, this indicates the interaction between **23** and I38T is not as stable as with WT PA_N_. E23K incubated with **23** showed a Δ*T*_M_ of 10.2 °C at 200 μM and 14.3 °C at 1 mM, ∼ 8 °C lower than with WT and 2 °C lower than the value obtained with I38T. This indicates the interaction between **23** and E23K is not as stable as WT or I38T. The difference between the Δ*T*_M_ values for **23** with E23K and I38T (>2 °C) is relatively small, suggesting that binding of **23** to these two mutants might be rather comparable, which is consistent with the SpS experiments (Table 1). The A36V mutant with compound **23** shows a trend similar to that of BXA with the Δ*T*_M_ being comparable to that of the WT, suggesting the binding of compound **23** to A36V is also largely unaffected by this mutation.

### X-ray Crystallography

To determine the mode of binding of BXA and compound **23** with WT, I38T, E23K, and A36V constructs, the complexes were crystallized, and the co-crystal structures determined. In addition to the co-crystal structures, the apo structures of WT, I38T, and E23K PA_N_ endonuclease were determined. In all apo structures, the active site metal cations Mn^2+^ are coordinated by His41, Glu80, Asp108, Glu 119, and Ile120 and a total of five water molecules, producing an octahedral coordination geometry at each metal center (Figure 1). No major structural changes were observed in the I38T apo structure compared with apo WT (Figure S2). When the apo structure of E23K is compared with that of WT, the terminus of α2, where the E23K mutation is located, is distorted (Figure S2). In WT PA_N_, Glu23 forms hydrogen bonds with Arg84, stabilizing the terminus of α2 which promotes the correct position of Tyr24 for base stacking. In the E23K structure, the change in charge that accompanies the change from a glutamate to a lysine results in a loss of hydrogen bonding with Arg84 and no stabilization. Lys23, Tyr24, and Arg84 become more flexible, causing disorder of α2 (Figure 3).

**Figure 2:**
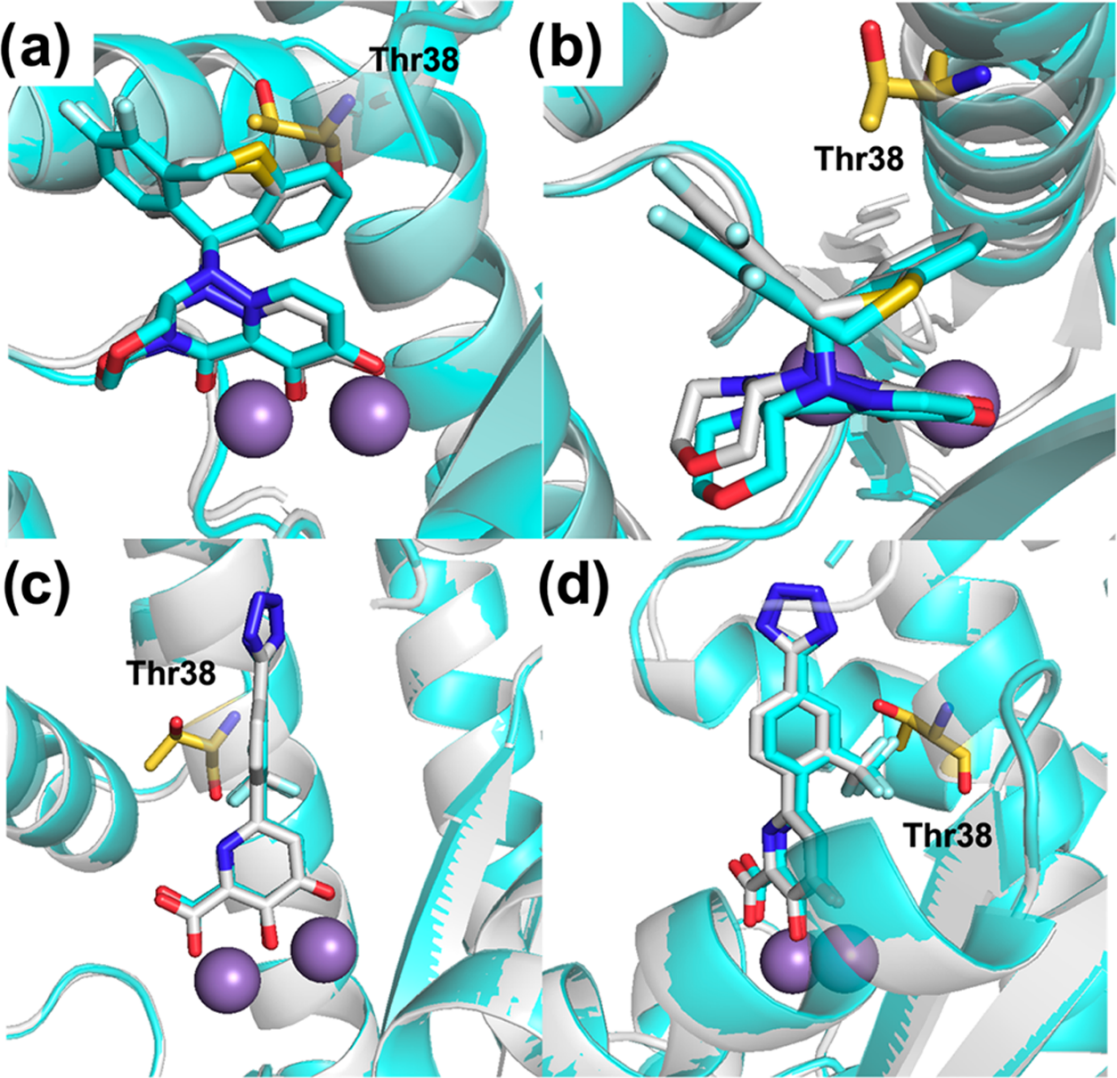
(a) Co-crystal structure of WT PA_N_ (gray PDB: 6FS6) overlaid with I38T mutant (cyan, Thr38 in yellow PDB: 8T5W) with BXA. (b) Alternate view of the co-crystal structure of WT PA_N_ (gray) overlaid with I38T mutant (cyan) with BXA (RMSD: 0.8 for pocket residues). The binding pose of BXA to I38T is virtually unaffected when compared to WT. Although BXA binding is largely unchanged, Thr38 must undergo a large rotamer change to accommodate BXA. (c) Co-crystal structure of WT PA_N_ (gray) overlaid with I38T mutant (cyan) with **23**. (d) Alternate view of the co-crystal structure of WT PA_N_ (gray) overlaid with I38T mutant (cyan) with **23** (RMSD: 0.2 for pocket residues). The change in binding of compound **23** with the I38T mutant shows a slight change in binding angle of the compound toward Thr38 so that the trifluoro group makes similar van der Waals contacts to that observed in the WT enzyme. The protein backbone (WT gray and I38T cyan) is shown as a cartoon, and Mn^2+^ ions are shown as purple spheres.

Upon binding either inhibitor, three water molecules are displaced and the octahedral geometry is maintained at each metal center for all constructs for which structures were obtained. BXA binds to the active site metal ions through a triad of oxygen donor atoms, including oxygen donor atoms from the ketone and carboxylic acid groups, while the “butterfly” shape of the remainder of the molecule provides extensive interactions with hydrophobic pockets of the active site in WT PA_N_ endonuclease (Figure 2). Significant interactions contributing to the high affinity of BXA include hydrophobic interactions between the aromatic rings and Ile38, hydrogen bonding between the hydroxyl group of Tyr24 and the BXA morpholine oxygen, as well as pi-stacking between the fluorinated BXA ring and Tyr24. Extended hydroxypyridinone heterocycle inhibitors including compound **23** coordinate to the active site metal ions with good affinity through hydroxypyridinone MBPs, and make two types of additional contacts with WT PA_N_.^19^ The addition of a 2′- or 3′-substituted benzene ring and a 4′-tetrazole drives the affinity for PA_N_ even lower. Compound **23** was previously reported to have an IC_50_ of 47 pM against WT PA_N_ endonuclease in an enzyme-based assay, making contacts with the active site wall via a 2′-trifluoro-substituted benzene ring, a weak van der Waals interaction with Ile38, and a 4′-tetrazole engaged in favorable hydrogen-bonding interactions shown to be critical in RNA binding with Lys34 and Arg124 (Figure 2).

The I38T mutation has been the most extensively studied mutation with several reported co-crystal structures with bound inhibitors (e.g., bound to RO-5, a BXA analogue).^12−14^ The reduced susceptibility of BXA for the I38T mutant has been attributed to less favorable van der Waals packing and induced fit changes.^6^ The butterfly shape of BXA provides near-perfect packing with hydrophobic pockets of the active site of WT PA_N_. Ile38 packs directly behind the V-shaped difluoro-dihydro-dibenzothiepine tail group, and this packing is slightly less effective with the change to the more polar, smaller Thr38. When comparing the BXA bound structure and the apo structure of I38T, a large rotamer change in Thr38 is observed (Figure S3). This rotamer change can also be observed when comparing the position of Thr38 in structures of BXA (Figure 2a) and **23** (Figure 2c) where the hydroxyl of Thr38 in the BXA structure is pointing toward the protein backbone and the hydroxyl of Thr38 in the structure of compound **23** is pointing toward the compound. No major local distortions are observed in α3 surrounding I38T in either structure (Figure S3). The change in the binding of compound **23** with the I38T mutant shows a slight change in the binding angle of the compound toward Thr38. The change to threonine removes a methyl group, making the slight tilt in **23** necessary so that the trifluoro group makes similar van der Waals contacts to that observed in the WT enzyme (Figure 2). However, small changes from the optimal binding angle are known to distort the octahedral coordination geometry around the metal centers and cause a decrease in affinity.^25,26^ The observed change in *K*_d_ and Δ*T*_M_ is most likely due to this sacrifice in favorable, highly enthalpic metal-binding interactions when the interaction with I38T is maintained. No rotamer changes were observed in Thr38 upon binding of compound **23**, unlike upon binding of BXA.

**Figure 3:**
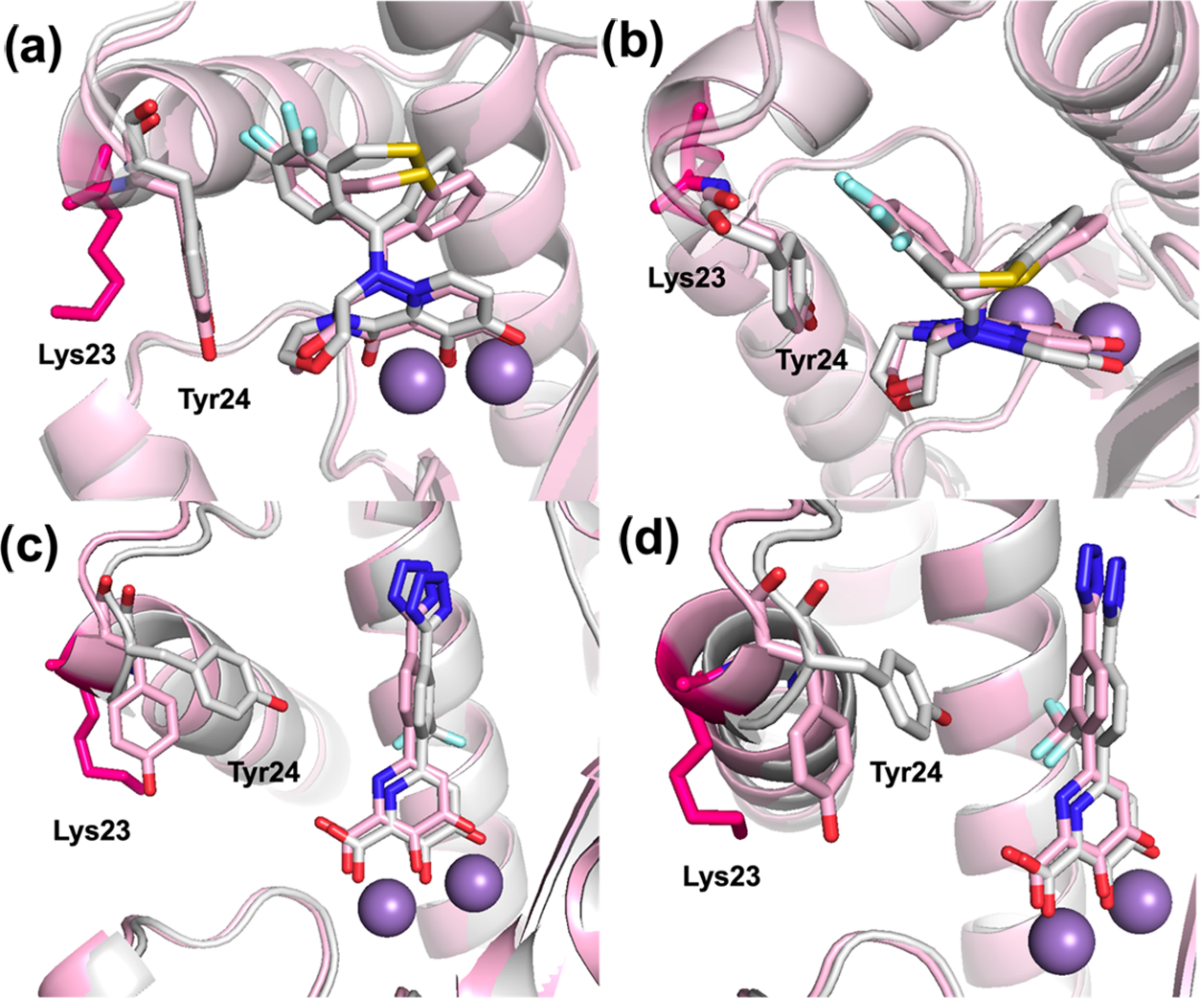
(a) Co-crystal structure of WT PA_N_ (gray PDB: 6FS6) overlaid with E23K mutant (pink, Lys23 in bright pink PDB: 8T6Z) with BXA coordinated to the Mn^2+^ ions. (b) Alternate view of WT PA_N_ (gray) overlaid with E23K mutant (pink) with BXA (RMSD: 0.9 Å for pocket residues). The E23K mutant shows a distortion in the terminus of α2 due to loss of stabilizing hydrogen-bonding interactions, which allows Tyr24 further into the active site causing a shift in BXA binding by 1.1 Å and **23** binding by 0.3 Å. (c) Co-crystal structure of WT PA_N_ (gray PDB: 8T94) overlaid with E23K mutant (pink PDB: 8T81) with **23**. (d) Alternate view of the co-crystal structure of WT PA_N_ (gray) overlaid with E23K mutant (pink) with **23** (RMSD: 0.3 Å for pocket residues). In this structure, differences in pi–cation interactions involving the 2′-trifluoro-substituted benzene ring and subsequently the position of the 4′-tetrazole are impacted. The ring is rotated in this structure to maintain the pi–cation interaction in the new position of Tyr24. The protein backbone (WT gray and E23K pink) is shown as a cartoon, and Mn^2+^ ions are shown as purple spheres.

From the biophysical assay data, it was expected that changes in BXA binding would be less significant with the E23K mutant when compared to the I38T mutant and there would be very slight, if any change at all in the binding of BXA with A36V. This was indeed the case, but significant changes to the position of Tyr24 and pi–cation interactions were observed. In WT PA_N_, Tyr24 makes a crucial base stacking interaction for RNA recognition in the active site. The RNA bases stack with Tyr24 and forms hydrogen-bonding interactions with Glu26 that, in turn, forms a salt bridge with Lys34.^15,24^ Glu23 contributes to this complex by hydrogen bonding with Arg84 and stabilizing the terminus of α2 and promoting the correct position of Tyr24 for base stacking.^15^ Comparing the co-crystal structures of WT and E23K with BXA bound, E23K disrupts the end terminus of α2 and allows Tyr24 to extend further into the active site, forcing BXA closer to α3 (Figure 3). The shift in BXA toward α3 disrupts hydrogen bonding between the hydroxyl group of Tyr24 and the BXA morpholine oxygen increasing the distance from 4.3 to 4.8 Å, as well as pi-stacking between the fluorinated BXA ring and Tyr24, increasing the distance from 5.2 to 6.3 Å. The change in position of BXA away from Tyr24 increases the bond distances, decreasing the strength of both the hydrogen bonding and pi-stacking (Figure 3). Unlike the BXA binding to I38T, no rotamer changes were found when comparing the BXA bound and unbound in the E23K structure. The disordered terminus of α2 and the change in position of Tyr24 could explain the relative change in binding affinity being greater for this mutant than for the I38T mutant. In solution, Lys23, Tyr24, and Arg84 are free to move and make a wide variety of transient protein–protein contacts and protein–inhibitor contacts that could lead to the destabilization of the binding of BXA without greatly affecting the overall protein stability. The E23K mutant in complex with compound **23** shows interesting changes in Lys23 and Tyr24 that have clearly defined electron density. In this structure, differences in pi–cation interactions involving the 2′-trifluoro-substituted benzene ring and subsequently the position of the 4′-tetrazole are impacted. The ring is rotated by 0.3 in this structure to maintain the pi–cation interaction in the new position of Tyr24 (Figure 3). This rotation causes changes in hydrogen bonding in the 4′-tetrazole with Lys34 and Arg124. There is also a slight change in MBP coordination, causing distortions from optimal octahedral geometry (Figure 3).

**Figure 4:**
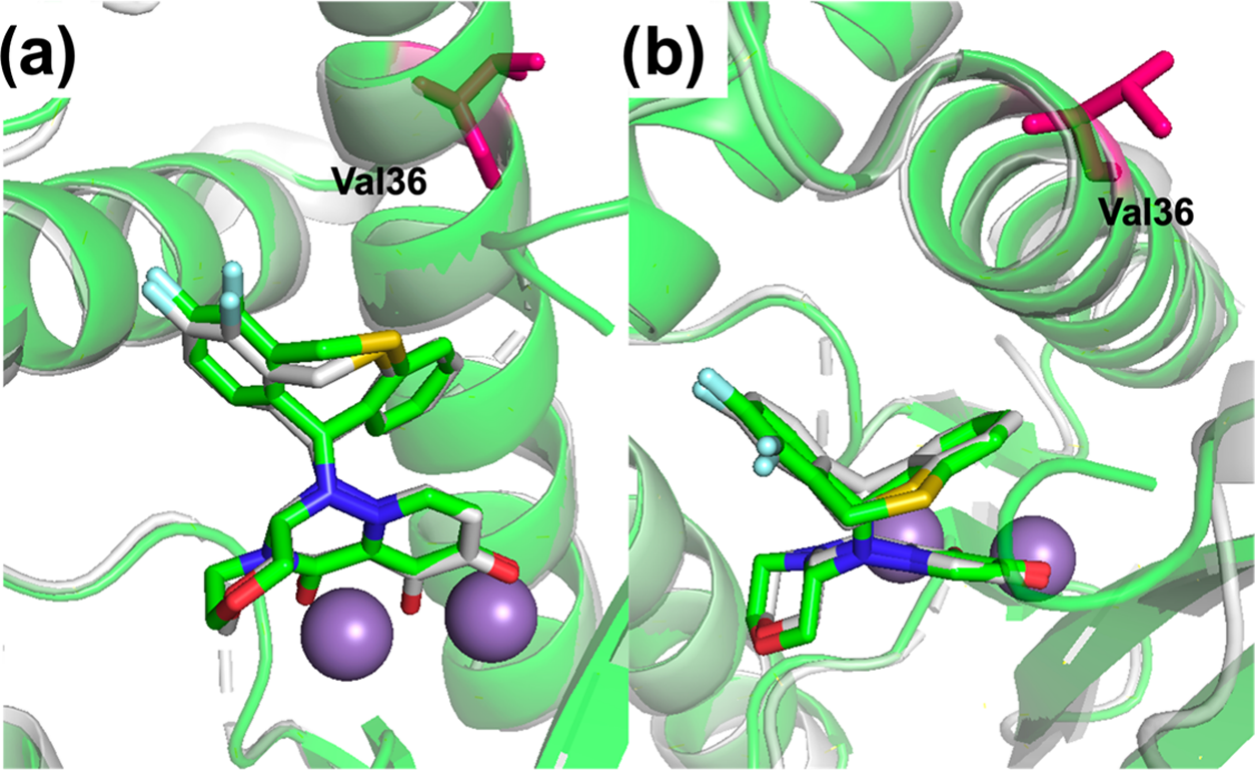
(a) Co-crystal structure and overlay of BXA bound to WT (gray PDB: 6FS6) and A36V mutant (green, Val36 in bright pink PDB: 8T5 V) PA_N_. (b) Alternate view of co-crystal structure and overlay of BXA bound to WT (gray) and A36V mutant (green) PA_N_. The structure of A36V in complex with BXA shows essentially no changes in the binding conformation of the inhibitor.

Although it was hypothesized that the A36V mutant would disrupt BXA binding by disrupting α3, the DSF experiments revealed that the A36V treated with BXA did not show a significant change in Δ*T*_M_. This suggests that the A36V mutation causes very minimal or no change at all to the binding of BXA. The structure of A36V in complex with BXA indeed shows no notable changes in the binding mode of BXA to the protein when compared to WT (Figure 4). No large changes to α3 such as rotamer changes, or changes to the overall structure of the helix were observed (Figure 4). The change from alanine to valine does change the side chain from a methyl to an isopropyl which changes both the conformational entropy and hydrophobicity of α3.^27^ A36V is partially surface exposed so it is possible the mutation promotes local or global unfolding.^28^ If this is the case, the crystal structure of A36V would be very similar to the WT structure, and assays such as DSF which measure unfolding through reporter binding to hydrophobic residues would give lower values than the WT due to A36V promoting unfolding of the protein. Additional studies to determine the Δ*G* between the unfolded and folded states would be needed to unambiguously determine if the resistance mechanism for this mutation is linked to the instability of the protein.^29^ Attempts to grow crystals of A36V bound to compound **23** were not successful. This is likely because compound **23** does not sufficiently stabilize the A36V mutant (Table 1) to enable crystallization.

## Materials and Methods

### Mutation Generation

Point mutations were generated in the PA_N_ endonuclease pET 28a plasmid by QuikChange mutagenesis (Agilent). Each PCR reaction of 50 μL contained 50 ng of template, 125 ng of primer pair, 200 μM dNTPs, and 3 units of Pfu DNA polymerase. The PCR cycles were initiated at 95 °C for 1 min to denature the template DNA, followed by 12 amplification cycles. Each amplification cycle consisted of 95 °C for 50 s, 60 °C for 1 min, and 68 °C for 6 min. The PCR cycles were finished with an extension step at 68 °C for 7 min. The PCR products were treated with 5 units of *Dpn*I at 37 °C for 1 h and transformed into ultra-competent cells and plasmid extracted. All mutations were first verified by Sanger Sequencing (Eton Biosciences) and then full, Non-Sanger plasmid sequencing (Primordium Laboratories) to ensure no other mutations occurred during the QuikChange prior to expression and purification.

### Protein Expression and Purification

Expression and purification of PA_N_ endonuclease were performed with slight modification (J. Med. Chem. 2019, 62, 9438–9449) and described in detail in the Supporting Information. Expression was induced by the addition of IPTG to a final concentration of 0.1 mM. The cultures were grown with vigorous shaking (250 rpm) overnight at room temperature. After ∼18 h, the cells were harvested by centrifuging at 2000*g* for 30 min at 4 °C. The resulting paste was stored at −80 °C prior to lysis.

### Protein Crystallography

Purified protein for crystallization was stored at 2.2–4.3 mg/mL at −80 °C after flash freezing in buffer consisting of 150 mM sodium chloride, 20 mM HEPES (pH 7.5), 2 mM MgCl_2_, and 2 mM MnCl_2_. Co-crystallization and crystal soaking methods were used to obtain co-crystal structures of inhibitors bound to PA_N_ endonuclease. BXA was purchased from Fisher and used without further purification. Compound **23** was previously synthesized according to a prior procedure (J. Med. Chem. 2019, 62, 9438–9449). For co-crystallization, protein was incubated with 0.5 mM inhibitor for 1 h on ice prior to setting the crystallization drops. For crystal soaking, fully formed holo crystals were transferred to a new drop containing 5 μL of reservoir solution and 1 μL of a 50 mM DMSO inhibitor stock solution (final concentration 8.3 mM). Crystals were left undisturbed overnight and either stored in liquid nitrogen or collected on an in-house X-ray diffractometer the following day. In both crystallization methods, crystals were grown using hanging drop and set in 24-well pregreased plates (Hampton HR3-171) with siliconized glass slides (Hampton HR3-231). A 5:1 ratio of purified protein to reservoir solution at room temperature was found to be the optimal ratio for the largest crystal formation. Reservoir solution consisted of 22–34% PEG (*M*_W_ = 4000 g/mol), 100 mM Tris (pH 8.35), and 220 mM sodium acetate. Colorless crystals with hexagonal bipyramidal morphology appeared within 2 days and reached full size after 1–2 weeks. Crystals were typically 50–200 μm in diameter. Crystals were cryoprotected with perfluoroether (Hampton HR2-814) prior to flash freezing in liquid nitrogen. Crystals were stored in liquid nitrogen until data collection.

### Biophysical Assays

DSF experiments were performed identically to (J. Med. Chem. 2019, 62, 9438–9449) and described in detail in the Supporting Information. SpS experiments were performed following manufacturer’s recommendation with slight modification. Protein was labeled using the Monolith X Protein Labeling Kit RED-NHS second Generation (NanoTemper Technologies, MO-L011) following the recommended procedure by the manufacturer. Labeled protein was frozen in 50 μL aliquots and stored at −80 °C to be used in future experiments. Prior to experiments, aliquots were thawed on ice and centrifuged for 10 min at 20 °C and 13 000 rpm to remove protein aggregates. Serial dilutions were prepared in assay buffer (1× MST buffer 50 mM Tris pH 7.4, 150 mM NaCl, 10 mM MgCl2, 0.05% Tween- 20, and 10% DMSO for BXA and 1X PBS pH 7.4 (Thermo Fisher, J62036-K7) with 0.1% pluronic F-127 for compound 23) in 384-well plates (Greiner) Reactions were initialized by gently mixing 20 μL of 40 nM protein to yield a final 1:1 solution and a volume of 40 μL per reaction. The reaction mixture was incubated on a plate shaker at room temperature, protected from light, at 300 rpm for 30 min. The reaction mixtures were loaded into premium capillaries (NanoTemper Technologies, MO-K025) and analyzed by Monolith X between 60 and 80% power. Compound **23** showed a ligand-induced fluorescent change and required an SD test to confirm the change in fluorescence. The SD test was performed according to the manufacturer’s instructions, and the remaining reaction after the run was used for the test.

## Conclusions

This work describes the first crystal structures of BXA in complex with E23K and A36V PA_N_ mutants and additional structures of BXA with WT and I38T. This study also examines another potent PA_N_ endonuclease inhibitor (**23**). Compound **23** has been previously reported to make hydrophobic interactions with Ile38 and could be susceptible to active site mutations and loss of inhibition similar to BXA. New information regarding the consequences of these mutations on inhibitor binding to PA_N_ endonuclease has revealed disruptions in crucial hydrophobic contacts, base stacking, and pi–cation interactions. The changes to active site binding of BXA with the I38T mutant have been replicated in this truncation model, and the weaker binding of BXA is attributed to less favorable van der Waals packing and induced fit changes. The change in binding of compound **23** with I38T was minimally disturbed, and only a small change in the position of the inhibitor in the active site was observed. In the E23K construct, Lys23, Tyr24, and Arg84 are more flexible and lead to destabilization of the binding of BXA without affecting the overall stability of the construct. Changes to the binding affinity of compound **23** with E23K were smaller, resulting in changes to the binding angle and changes in hydrogen bonding. E23K disrupts the end terminus of α2 and allows Y24 to extend further into the active site, disrupting important hydrogen-bonding, pi–cation, and base stacking interactions made by inhibitors or natural substrates such as RNA. E23K was found to interrupt hydrogen-bonding and cation interactions upon binding of both BXA and **23**. The A36V mutant melts at around 10 °C lower than its WT, I38T, and E23K counterparts. Despite this, A36 V treated with BXA did not have a significant change in Δ*T*_M_. This suggested that the A36V mutation makes the endonuclease unstable and causes very minimal, or no change at all, to the binding of BXA. The structure of A36V with BXA was obtained and indeed showed no notable changes in the binding mode of BXA to the protein when compared to BXA binding to WT. Overall, the findings here may prove useful for the design of more efficient inhibitors against these resistant mutants by providing new insight into what drives resistance.

## Supporting Information Available

Generation of mutants, protein expression, purification, and crystallographic details (including crystallography tables and electron density maps); detailed experimental procedure for differential scanning fluorimetry and spectral shift/microscale thermophoresis (Figures S1–S8) (Tables S1–S3) (PDF)

## Accession Codes

The UNIPROT entry for the N-terminal domain of the RNA-dependent RNA polymerase, PA_N_ is C3W5S0.

## Abbreviations

FDA: Food and Drug Administration
IC_50_: half-maximal inhibitory concentration
PA_N_: N-terminal domain of the RNA-dependent RNA polymerase
SAR: structure–activity relationship
BXM: Baloxavir Marboxil
BXA: Baloxavir Acid
NPI: nonpharmaceutical intervention
NAI: neuraminidase inhibitor
MBP: metal-binding pharmacophore
WT: wild type
*K*_d_: dissociation binding constant
*T*_M_: thermal melting
SpS: spectral shift
FC: fold change
