# Supplementary material for: Structural Studies of Inhibitors with Clinically Relevant Influenza Endonuclease Variants
Source: Biochemistry. 2024 Jan 8;63(3):264–72. doi: 10.1021/acs.biochem.3c00536 (PMC10851415; doi:10.1021/acs.biochem.3c00536)
Supplement: Supplementary file 1 — bi3c00536_si_001.pdf [file bi3c00536_si_001.pdf]

Supporting Information

Structural Studies of Inhibitors with Clinically-relevant Influenza Endonuclease Variants

Alysia J. Kohlbrand,[a] Ryjul W. Stokes,[a] Banumathi Sankaran,[b] and Seth M. Cohen [a]*

[a] Department of Chemistry and Biochemistry, University of California, San Diego, 9500 Gilman Drive, La Jolla, CA 92093, United States.

[b] The Berkeley Center for Structural Biology, Advanced Light Source, Lawrence Berkeley National Laboratory, Berkeley, California, 94720, United States

*scohen@ucsd.edu

Table of Contents

Mutation Generation S2

Protein Expression and Purification S2

Differential Scanning Fluorimetry S5

Spectral Shift and Microscale Thermophoresis S6

Protein Crystallography S6

Apo Crystal Structures S7

Protein Crystallography Tables S8

Protein Crystallography Electron Density Maps S12

Mutation Generation

Point mutations were generated in the PA_N endonuclease pET 28a plasmid by QuikChange mutagenesis (Agilent). Each PCR reaction of 50 µl contained 50 ng of template, 125 ng primer pair, 200 µM dNTPs and 3 units of Pfu DNA polymerase. The PCR cycles were initiated at 95°C for 1 min to denature the template DNA, followed by 12 amplification cycles. Each amplification cycle consisted of 95 °C for 50 sec, 60 °C for 1 min, and 68 °C for 6 min. The PCR cycles were finished an extension step at 68 °C for 7 min. The PCR products were treated with 5 units of *Dpn*I at 37°C for 1 h and transformed into ultra competent cells and plasmid extracted. All mutations were first verified by Sanger Sequencing (Eton Biosciences) and then full, Non-Sanger plasmid sequencing (Primordium Labs) to ensure no other mutations occurred during the QuikChange prior to expression and purification.

Protein Expression and Purification

Expression and purification of PA_N endonuclease was performed as reported previously (*J. Med. Chem.* **2019,** *62*, 9438-9449). The pandemic isolate A/California/04/2009(H1N1) N-terminal PA (PA_N) endonuclease Δ52-64:Gly truncated construct was expressed from a pET-28a parent vector containing a kanamycin-resistance reporter gene with expression inducible by the Lac1 operon. PA_N endonuclease was expressed as an 8-histidine tagged fusion protein cleavable by TEV protease. The transformation protocol was adapted from pET system manual (Novagen) using single competent BL21 cells. Briefly, 1 µL of 25 ng/µL recombinant plasmid was used for transformation. Cells were mixed with the plasmid and were heat shocked at 42 °C for 30 sec followed by incubation on ice for 3 min. Outgrowth was plated on LB agarose plates containing 50 µg/mL kanamycin and was incubated overnight at 37 °C. One colony was scraped from the LB plate and added to 5 mL of SOC broth containing 50 µg/mL kanamycin and was incubated overnight at 37 °C with shaking at 125 rpm. SOC media (100 mL) containing 50 µg/mL kanamycin was combined with the 5 mL overnight growth and was incubated with shaking at 200 rpm at 37 °C until the OD_{600} of this starter culture reached >2 (3-4 h). The culture was equally divided into 6, 2L flasks containing 1L of expression media (TB media with added 0.2% dextrose, 0.1 mM $MnCl_2$, and 0.1 mM $MgSO_4$, 50 µg/mL kanamycin). Cells were grown to and OD_{600} between 0.4-0.6 at room temperature with shaking at 200 rpm (3-4 h). Expression was then induced by addition of IPTG to a final concentration of 0.1 mM. The cultures were grown with vigorous shaking (250 rpm) overnight at room temperature. After ~18 h the cells were harvested by centrifuging at 2000g for 30 min at 4 °C. The resulting paste was stored at -80 °C prior to lysis.

The cell paste was thawed on ice for 2 h and resuspended in 25-35 mL of lysis buffer (1% Triton-X, 1mM $MgCl_2 \cdot 6H_2O$, 2mM DTT, 10-100 µg/mL DNAse-1, 1 mg/ml lysozyme, and 1% glycerol) and EDTA free protease inhibitor (Roche). Cells were lysed using a probe sonicator (Fisherbrand model 120) with cycles of 25 sec pulses and 59 sec rest at 60% amplitude. Cell debris was then pelleted by centrifugation at 10000 rpm for 45 min at 4 °C. The supernatant was decanted from the pellet, and a HisTrap FF (Cytiva) column was utilized to isolate His-tagged fusion protein from the cell lysates according to the manufacturer's recommendations at 4 °C. Briefly, cell-free lysates were loaded on 5 mL column that had previously been charged with Ni ions. The column was then washed with binding buffer (20 mM Na_2PO_4, 500 mM NaCl, 25 mM imidazole, pH 7.4) until fraction absorbance reached a steady baseline. The protein was then eluted over a gradient from 0-100% elution buffer (20 mM Na_2PO_4, 500 mM NaCl, 500 mM imidazole, pH 7.4) at a flow rate of 4 mL/min. PA_N endonuclease eluted between 40-60% elution buffer. SDS-PAGE analysis showed a band corresponding to PA_N endonuclease running at ~23 kDa with several small impurities.

Fractions containing PA_N endonuclease were combined in a 10K MWCO dialysis bag with 1000 units of TEV protease and were dialyzed against dialysis buffer (100 mM NaCl, 1 mM dithiothreitol, 1 mM $MnCl_2$, 20 mM Tris, 5% glycerol, pH 8.0) overnight with three buffer exchanges. The proteolytic cleavage of the fusion protein is slow and greatly benefits from the addition of excess TEV protease. A white precipitate forms over time. The solution was run through the Histrap FF column equilibrated with the same binding buffer as before. The resulting flow through contained His-cleaved PA_N endonuclease, which was then concentrated to 5-10 mg/mL using a pressurized Amicon and/or spin Amicon concentrator. The concentrated protein was then purified on a gel-permeation size exclusion column (GE Superdex 75, 10/300 GL) according to manufacturer recommendations in buffer (150 mM NaCl, 2 mM $MgCl_2$, 2 mM $MnCl_2$, 20 mM HEPES, pH 7.5). A large peak corresponding to the cleaved PA_N endonuclease eluted at ~12 mL eluent. A small shoulder before the main peak was occasionally observed, which contained primarily uncleaved and/or unfolded PA_N endonuclease construct. Fractions containing pure cleaved PA_N endonuclease were combined and concentrated to 2-5 mg/mL. Stored protein was flash-frozen in liquid nitrogen and was kept at -80 °C. This protein was suitable for use in enzyme or thermal shift assays or for protein crystallography.

Differential scanning fluorometry (DSF)

Each well of a 96-well 0.2 mL optical MicroAmp (ThermoFisher) thermocycler plate contained a volume of 20 µL containing final concentrations of 1 µg PA_N endonuclease, 200 µM or 1 mM inhibitor, and 1×SYPRO orange Thermal Shift dye in buffer (150 mM NaCl, 2 mM $MnCl_2$, 20 mM HEPES pH 7.5) with 4% DMSO. A master mix containing PA_N endonuclease, 1×SYPRO orange Thermal Shift dye, and buffer was made and 16 µL added to each well. Either 1mM inhibitor stocks or 20% DMSO stocks were made, to which 4 µL was added to the plate to make a total of 20 µL final volume. Each well was mixed thoroughly by pipetting up and down, with care to prevent air bubbles from forming. The presence of this small concentration of DMSO was found to have a negligible effect on ΔT_M values of native PA_N endonuclease. Thermocycler plate wells were sealed prior to analysis, and the plate was then heated in a thermocycler from 25 to 99 °C at a ramp rate of 0.05 °C/sec. Fluorescence was read using the ROX filter channel (λ_{ex} = 580 nm; λ_{em} = 623 nm), and the fluorescence signal was fitted to a first derivative curve to identify TM. Native WT PA_N endonuclease was generally observed to melt with a T_M= 58−59 °C, I38T T_M= 58-59 °C, E23K T_M= 60-61 °C, and A36V T_M= 51-52 °C.

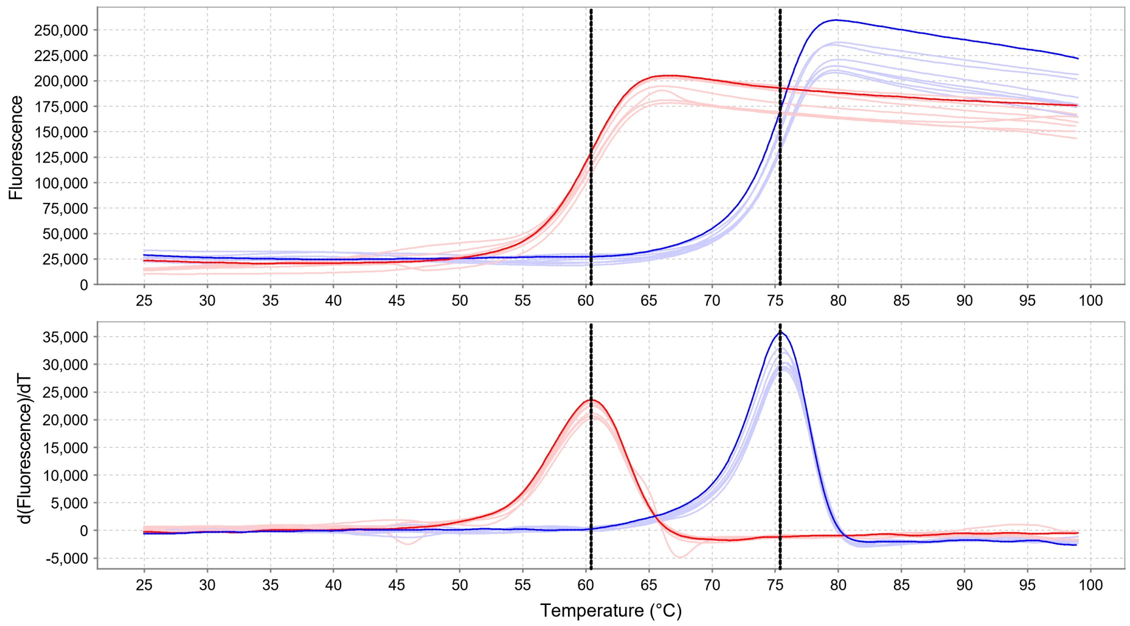

Figure S1. Representative thermal shift traces for I38T mutant with **BXA**. Fluorescent melting curve trace (*top*) and first derivative of the fluorescent trace (*bottom*). The maximum of the derivative peak for each trace is the melting point of the protein. Red traces are WT with **BXA** melting curve replicates, blue traces are I38T mutant with **BXA** melting curve replicates. A total of eight independent replicates were performed for each experiment.

Spectral Shift (SpS) and Microscale Thermophorsis (MST) Assay

Protein was labeled using the Monolith X Protein Labeling Kit RED-NHS 2nd Generation (NanoTemper Technologies, MO-L011) following the recommended procedure by the manufacturer. Labeled protein was frozen in 50 µL aliquots and stored at -80 °C to use in future experiments. Prior to experiments, aliquots were thawed on ice and centrifuged for 10 min at 20 °C and 13,000 RPM to remove protein aggregates.

Serial dilutions were prepared in assay buffer (1X MST buffer 50 mM Tris pH 7.4, 150 mM NaCl, 10 mM MgCl2, 0.05% Tween- 20, and 10% DMSO for BXA and 1X PBS pH 7.4 (Thermo Fisher, J62036-K7) with 0.1% pluronic F-127 for compound 23) in 384 well plates (Greiner) Reactions were initialized by gently mixing 20 µL of 40 nM protein to yield a final 1:1 solution and a volume of 40 µL per reaction. The reaction was incubated on a plate shaker at room temperature, protected from light, at 300 RPM for 30 min. The reaction mixtures were loaded into premium capillaries (NanoTemper Technologies, MO-K025) and analyzed by the Monolith X between 60 and 80% power.

Compound **23** showed a ligand-induced fluorescent change and required an SD test to confirm the change in fluorescence. The SD test was performed to the manufacture's instructions and the remaining reaction after the run was used for the test.

For a step-by-step explanation of the process used here please refer to *Bio-protocol* **2020,** *10*, e3574-e3574.

Protein Crystallography

Purified protein for crystallization was stored at 2.2-4.3 mg/mL at -80 °C after flash freezing in buffer consisting of 150 mM sodium chloride, 20 mM HEPES (pH 7.5), 2 mM $MgCl_2$, and 2 mM $MnCl_2$. Co-crystallization and crystal soaking methods were used to obtain co-crystal structures of inhibitors bound to PA_N endonuclease. BXA was purchased from Fisher and used without further purification. Compound **23** was previously synthesized according to a prior procedure (*J. Med. Chem.* **2019,** *62*, 9438-9449). For co-crystallization, protein was incubated with 0.5 mM inhibitor for 1 hour on ice prior to setting the crystallization drops. For crystal soaking, fully formed holo crystals were transferred to a new drop containing 5 µL of reservoir solution and 1 µL of 50 mM DMSO inhibitor stock solution (final concentration 8.3 mM). Crystals were left undisturbed overnight and either stored in liquid nitrogen or collected on an in-house X-ray diffractometer the following day. In both crystallization methods, crystals were

grown using hanging drop and set in 24-well pre-greased plates (Hampton HR3-171) with siliconized glass slides (Hampton HR3-231). A 5:1 ratio of purified protein to reservoir solution at room temperature was found to be the optimal ratio for the largest crystal formation. Reservoir solution consisted of 22-34% PEG (MW 4000 g/mol), 100 mM Tris (pH 8.35), and 220 mM sodium acetate. Colorless crystals with hexagonal bipyramidal morphology appeared within 2 days and reached full size after 1-2 weeks. Crystals were typically 50 to 200 microns in diameter. Crystals were cryoprotected with perfluoroether (Hampton HR2-814) prior to flash freezing in liquid nitrogen. Crystals were stored in liquid nitrogen until data collection.

Datasets for **E23K apo** and **E23K/BXA** were collected on an in-house X-ray diffractometer. For these experiments, diffraction data was collected at 100 K on a Bruker X8 Proteum diffractometer using a Bruker Microfocus Rotating Anode (MicroStar FR- 592) X-ray generator with a Bruker APEX II CCD detector at wavelength 1.54178 Å. Data was integrated, scaled, and merged using the Bruker APEX3 software package (Bruker, 2017). Datasets for the following structures, **I38T/BXA, I38T/23, and WT/23** were collected at the Advanced Light Source (ALS), Lawrence Berkeley National Laboratory, in collaboration with Dr. Banu Sankaran through the Collaborative Crystallography program. Datasets for **A36V/BXA, WT apo, and I38T apo** were collected at ALS on beamline 8.2.1. The dataset for **E23K/23** was collected at the Stanford Synchrotron Radiation Lightsource (SSRL), Stanford Linear Accelerator Center (SLAC) National Laboratory on beamline 12-1. For all datasets (including datasets collected on the in-house X-ray generator), phasing was determined by molecular replacement against a previously published PA_N endonuclease structure (PDB 8DDB) using PHASER. All structures were refined with Phenix version 1.19.2.

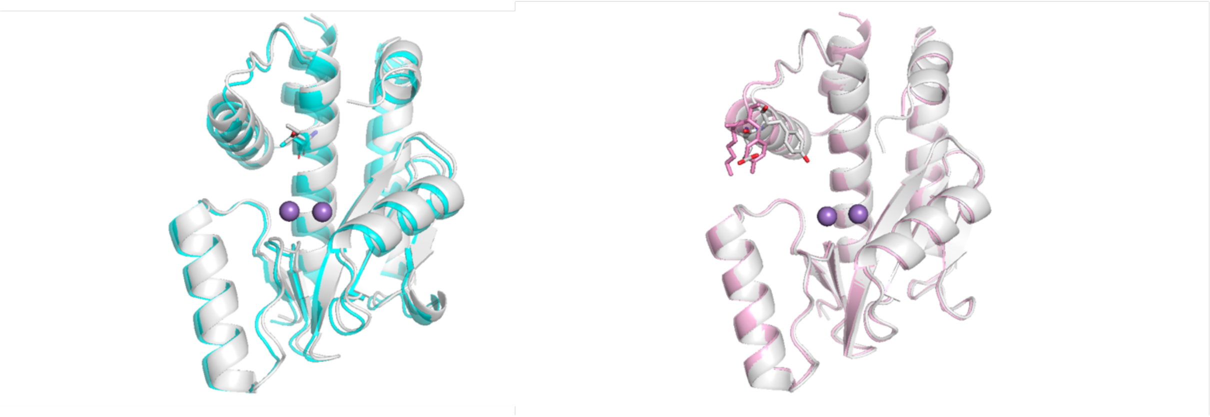

Figure S2. *Left*: Crystal structure of WT PA_N (gray) overlayed with I38T mutant (cyan). *Right*: crystal structure of WT PA_N (gray) overlayed with E23K mutant (pink). The protein backbone (WT gray, I38T cyan, and E23K pink) is shown as a cartoon and Mn^{2+} ions are shown as purple spheres.

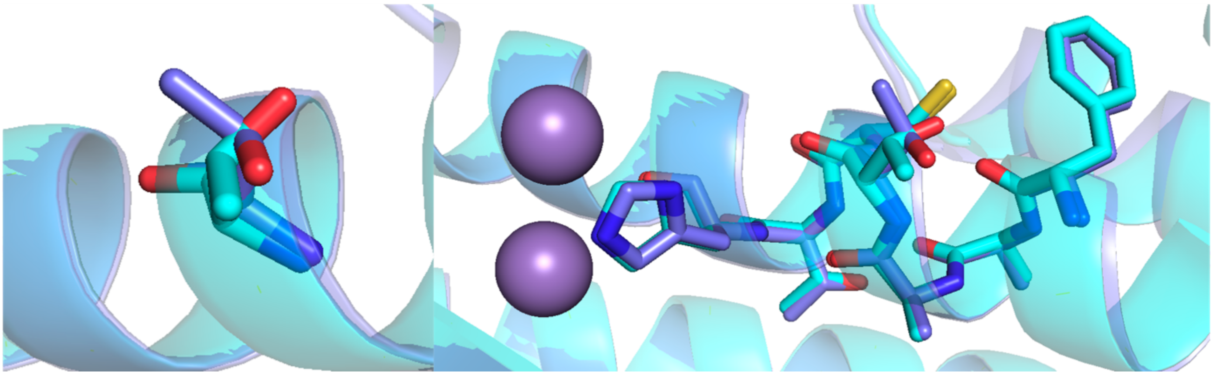

Figure S3. Crystal structure of apo I38T PA_N (purple) overlayed with I38T with **BXA** (cyan) with Mn^{2+} ions shown as purple spheres. *Left:* Close up of rotamer change of Thr38 observed during the binding of **BXA** to I38T mutant PA_N. *Right:* Alignment of adjacent residues to I38T (F35, A36, A37, T38, C39, T40, and H41). No significant local changes are observed in the residues surrounding Thr38. Thr38 must undergo a large rotamer change to accommodate the binding of **BXA** causing an almost 180° rotation of Thr38.

Table S1. X-ray crystallographic data collection and refinement statistics for the WT, I38T, and E23K apo structures and E23K with **23** bound.

Protein Construct	WT	I38T	E23K	E23K
Compound	-	-	-	**23**
**PDB**	**8T5Z**	**8T67**		**8VGZ**
**Data collection statistics**				
Resolution range (Å)	65.66-2.03 (2.10-2.03)	64.60-2.08 (2.12-2.08)	65.74-1.91 (1.98-1.91)	37.69-2.60 (2.69-2.60)
Space group	P 62 2 2	P 62 2 2	P 62 2 2	P 62 2 2
Cell dimensions a, b, c, (Å) α, β, γ (°)	75.82 75.82 122.262 90 90 120	74.589 74.589 119.534 90 90 120	75.915 75.915 120.631 90 90 120	75.3776 75.3776 121.533 90 90 120
Unique reflections	25379	22347	27414	6736
Completeness (%)	100.0 (100.0)	100.0 (100.0)	100.0 (99.8)	100.0 (99.9)
Mean I/sigma(I)	24 (1.8)	18 (2.2)	17 (2.3)	12 (1.7)
R-merge	0.070 (1.772)	0.091 (1.450)	0.078 (0.407)	0.021 (0.406)
R-measured	0.072 (1.816)	0.093 (1.486)	0.079 (0.413)	0.030 (0.574)
R-work	0.2220 (0.3052)	0.2061 (0.2879)	0.2278 (0.3933)	0.2443 (0.3710)
R-free	0.2478 (0.3447)	0.2481 (0.3396)	0.2660 (0.4126)	0.2954 (0.4047)
RMS(bonds)	0.003	0.005	0.009	0.011
RMS(angles)	0.56	0.82	1.12	1.58
Ramachandran favored (%)	98.27	97.67	95.38	97.65
Ramachandran outliers (%)	0	0	0	0
Average B-factor	55	58.7	63.7	71.4
Redundancy	25	25		25
CC1/2	1.000	0.999	0.999	1.000

* Metrics for highest resolution shell given in parentheses

Table S2. X-ray crystallographic data collection and refinement statistics for WT and I38T with **23** bound, and I38T and E23K with **BXA** bound.

Protein Construct	WT	I38T	I38T	E23K
Compound	**23**	**BXA**	**23**	**BXA**
**PDB**	**8T94**	**8T5W**	**8T6Z**	
**Data collection statistics**				
Resolution range (Å)	65.81-2.11 (2.16-2.11)	57.34-1.65 (1.71-1.65)	65.74-1.91 (1.98-1.91)	65.74-1.91 (1.98-1.91)
Space group	P 62 2 2	P 62 2 2	P 62 2 2	P 62 2 2
Cell dimensions a, b, c, (Å) α, β, γ (°)	75.986 75.986 120.791 90 90 120	75.111 75.111 121.444 90 90 120	75.915 75.915 120.631 90 90 120	75.979 75.979 121.005 90 90 120
Unique reflections	12825	25063 (2449)	27414	10783 (1053)
Completeness (%)	100.0 (100.0)	99.9 (100.0)	100.0 (99.8)	99.81 (99.15)
Mean I/sigma(I)	18 (1.7)	28 (2.0)	17 (2.3)	24.87 (2.54)
R-merge	0.085 (0.693)	0.054 (1.085)	0.078 (0.407)	0.08323 (2.05)
R-measured	0.086 (0.707	0.103 (0.815)	0.079 (0.413)	0.08492 (2.092)
R-work	0.2236 (0.3668)	0.2028 (0.3090)	0.2278 (0.3933)	0.1876 (0.3225)
R-free	0.2575 (0.3806)	0.2450 (0.3478)	0.2660 (0.4126)	0.2442 (0.4187)
RMS(bonds)	0.009	0.01	0.009	0.01
RMS(angles)	1.11	1.7	1.12	1.02
Ramachandran favored (%)	94.25	97.69	95.38	94.89
Ramachandran outliers (%)	0	0	0	0.57
Average B-factor	70.8	40.7	63.7	64.51
Redundancy				26.2
CC1/2	1.000	0.999	0.999	1.000

* Metrics for highest resolution shell given in parentheses

Table S3. X-ray crystallographic data collection and refinement statistics for A36V with **BXA** bound.

Protein Construct	A36V
Compound	**BXA**
**PDB**	**8T5V**
**Data collection statistics**	
Resolution range (Å)	64.71-1.79 (1.81-1.79)
Space group	P 62 2 2
Cell dimensions a, b, c, (Å) α, β, γ (°)	74.724 74.724 121.619 90 90 120
Unique reflections	16971 (2053)
Completeness (%)	100.0 (100.0)
Mean I/sigma(I)	17 (2.0)
R-merge	0.075 (1.745)
R-measured	0.077 (1.770)
R-work	0.2192 (0.4079)
R-free	0.2550 (0.4629)
RMS(bonds)	0.07
RMS(angles)	1.54
Ramachandran favored (%)	97.14
Ramachandran outliers (%)	0
Average B-factor	56.1
Redundancy	25
CC1/2	0.999

* Metrics for highest resolution shell given in parentheses

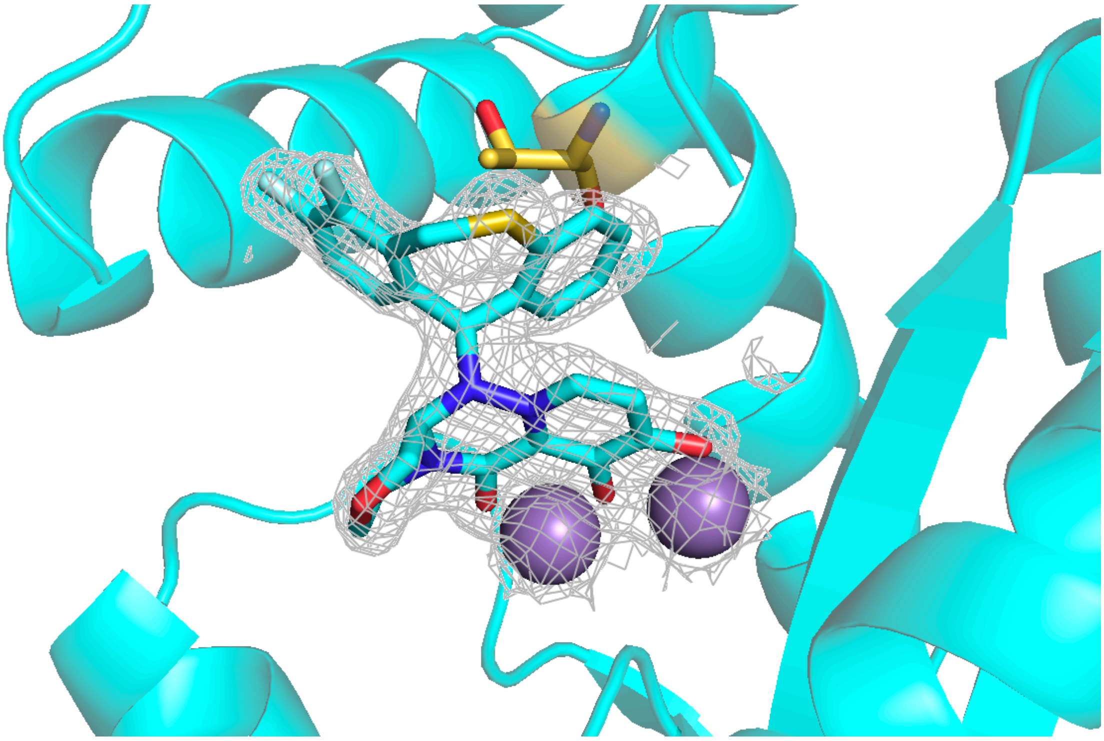

Figure S4. Co-crystal structure of **BXA** bound to the I38T mutant (cyan, Thr38 in yellow). The electron density of **BXA** is displayed as a gray mesh. Mesh is $2F_o$–F_c contoured at 1.8σ.

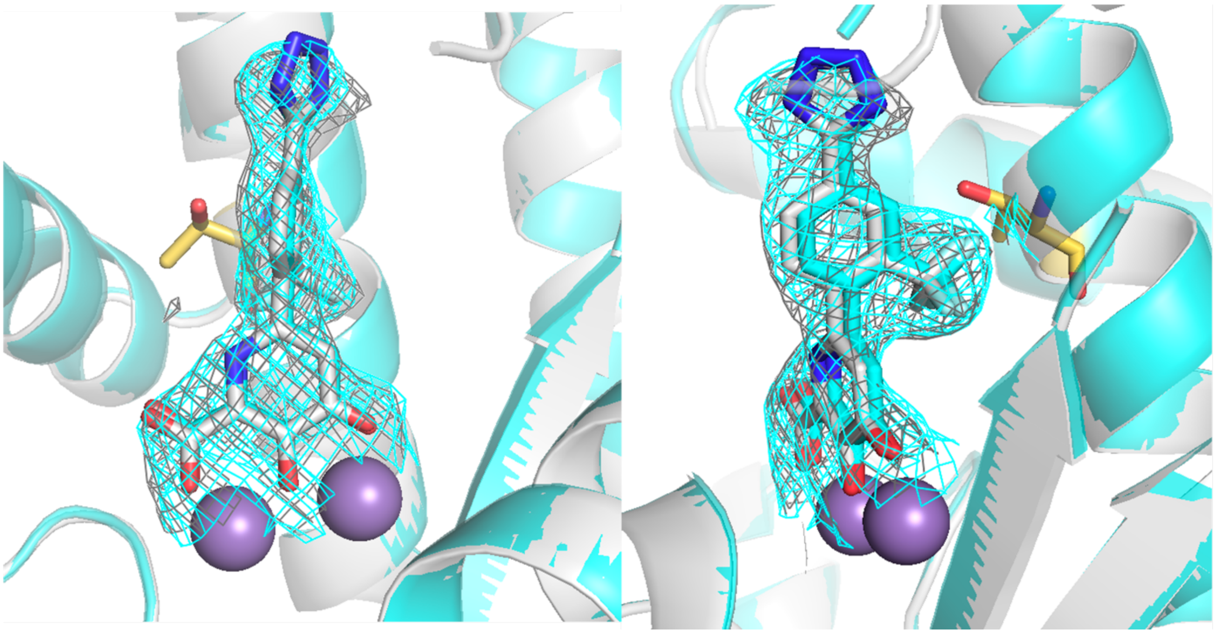

Figure S5. Co-crystal structure of **23** bound to WT PA_N (gray) overlayed with **23** bound to the I38T mutant (cyan, Thr38 in yellow). *Right:* Alternate view of the co-crystal structure of WT PA_N (gray) overlayed with I38T mutant (cyan) with **23**. The electron density of **23** is displayed as a mesh (WT in gray and I38T in cyan). Mesh is $2F_o–F_c$ contoured at 2σ. The change in binding of compound **23** with the I38T mutant shows a slight change in binding angle of the compound toward Thr38 so that the trifluoro group makes similar van der Waals contacts to that observed in the WT enzyme.

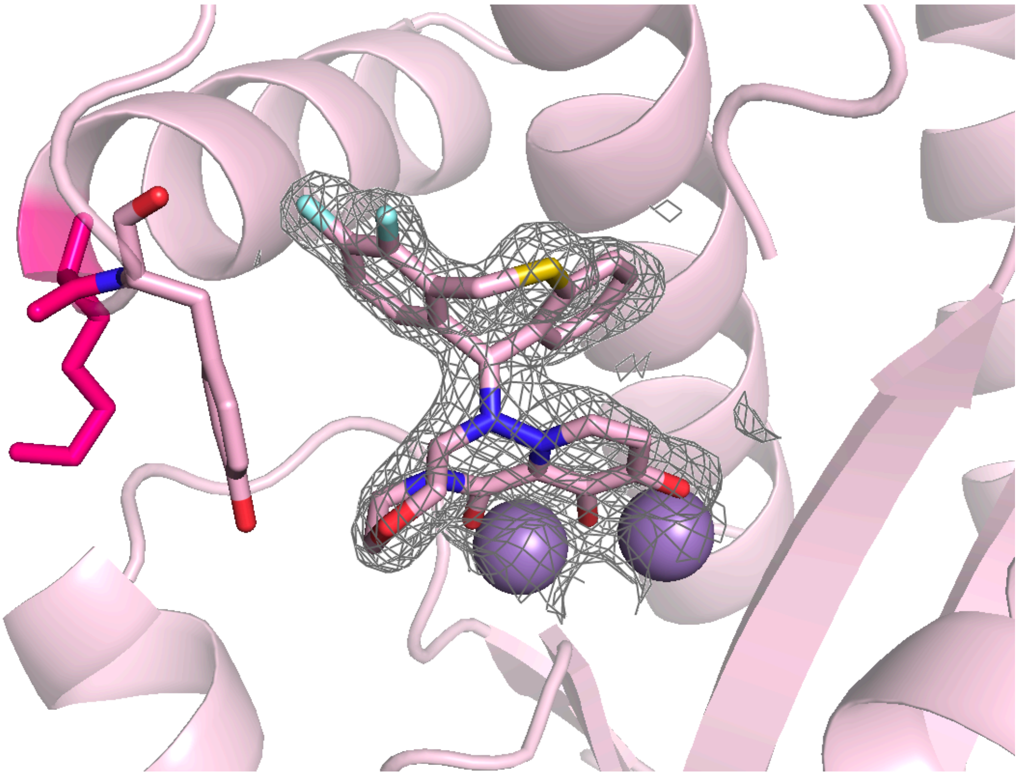

Figure S6. Co-crystal structure of **BXA** bound to the E23K mutant (E23K pink, Lys23 in bright pink). The electron density of **BXA** is displayed as a gray mesh. Mesh is $2F_o–F_c$ contoured at 1.8σ.

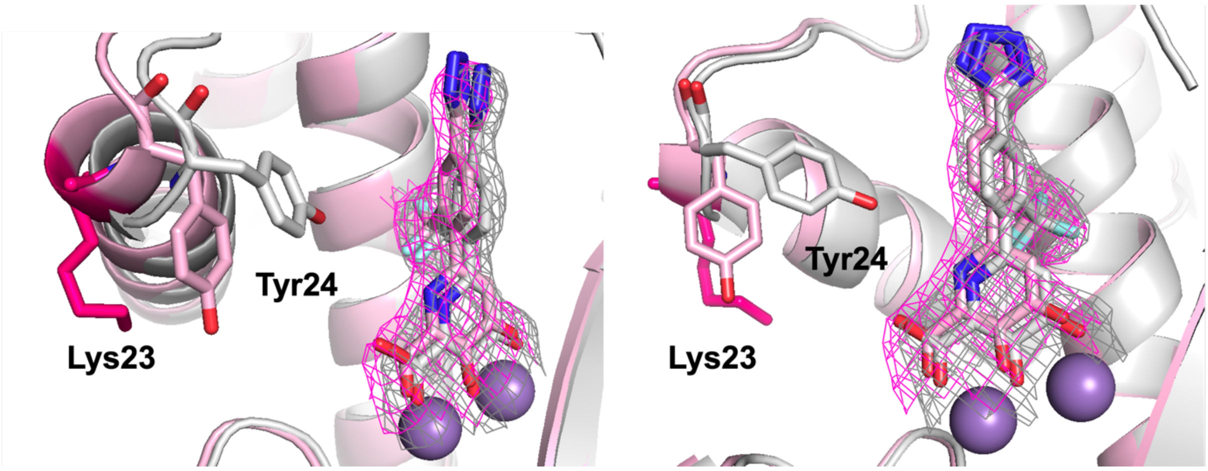

Figure S7. Co-crystal structure of **23** bound to the WT PA_N (gray) overlayed with **23** bound to the E23K mutant (pink, Lys23 in bright pink). *Right:* Alternate view of the co-crystal structure of WT PA_N (gray) overlayed with E23K mutant (pink) with **23**. The electron density of **23** is displayed as a mesh (WT in gray and E23K in Pink). Mesh is $2F_o–F_c$ contoured at 1.8σ. In this structure, differences in pi-cation interactions involving the 2′-trifluoro substituted benzene ring and subsequently the position of the 4′-tetrazole are impacted. The ring is rotated in this structure to maintain the pi-cation interaction in the new position of Tyr24.

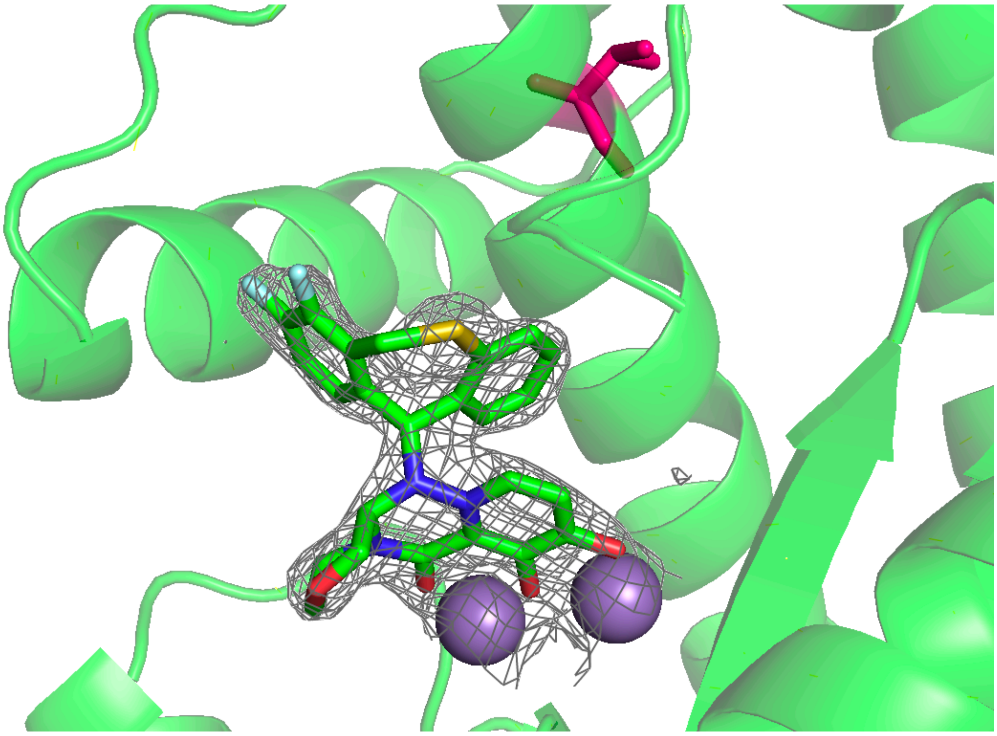

Figure S8. Co-crystal structure **BXA** bound to the A36V mutant (A36V green, Val36 in bright pink). The electron density of **BXA** is displayed as a gray mesh. Mesh is $2F_o$–F_c contoured at 1.8σ.
